# Patient Preference Studies During Early Drug Development: Aligning Stakeholders to Ensure Development Plans Meet Patient Needs

**DOI:** 10.3389/fmed.2019.00082

**Published:** 2019-04-24

**Authors:** Nigel S. Cook, Julie Cave, Anke-Peggy Holtorf

**Affiliations:** ^1^Novartis Pharma AG, Basel, Switzerland; ^2^Novartis Pharmaceuticals Corporation, East Hanover, NJ, United States; ^3^Health Outcomes Strategies GmbH, Basel, Switzerland

**Keywords:** patient preferences, patient-based evidence, multi-stakeholder dialog, drug development, health technology assessment (HTA), dry eye disease, social media listening, online bulletin board

## Abstract

Although patient preferences have been studied broadly for marketed products or around the time of submission to authorities and launch, patient preference studies have rarely been used during the early drug development phases. In this paper, we formulate three hypotheses supporting the use of patient preference studies in early product development: (1) integration of the patient perspective into the development process from phase 1 onwards will result in healthcare solutions with outcomes that best address patients' needs; (2) a structured process to build patient-based evidence involving partnerships between patients and other key stakeholders will improve alignment of development activities with the needs of patients; (3) quantitative patient preference research built on robust qualitative insights is necessary to strengthen development decisions in the interests of patients. To illustrate such a structured process, we describe qualitative insights research (social media analysis and online bulletin boards) and quantitative patient preference studies in dry eye disease and non-alcoholic steatohepatitis conducted during early product development by a pharmaceutical company to generate patient-based evidence. The outputs from such early patient preference studies are being used to inform patient reported outcome strategies, clinical development strategies, product design and delivery features, and form the basis for early dialog with regulators, health technology assessment (HTA) bodies and payers to ensure focus and alignment on patient-relevant endpoints. Furthermore, to discuss and theoretically substantiate our hypotheses, we review how different groups and organizations are working to embrace fully the patient perspective in product development and healthcare decision-making. The hypotheses are commensurate with the general trend toward patient-centered healthcare and the activities initiated by regulators, HTA agencies, and patient organizations. We advocate that all healthcare players should actively contribute to aligning on best practices concerning choice of methodologies and engage in multi-stakeholder dialog along the entire product development chain, to realize health technologies that best meet the needs of patients.

## Introduction

Pharmaceutical companies are increasingly recognizing the value of incorporating the patients' perspectives into the clinical development process to deliver treatments and outcomes that are relevant to them. Traditionally, clinical development has been weighted toward clinically held beliefs of what is important to patients, with the patients' perspective being captured only during the later stages of development either via patient reported outcomes (PROs) or anecdotal evidence. Recent evidence suggests that even in well-studied diseases, clinically held beliefs of what is important to patients may differ from the needs expressed by patients themselves (1, 2).

Companies who embrace the patient perspective early in the product design stage are most likely to ensure a fit of their product to the real needs of patients and provide the benefits patients are seeking (3). The optimal timing as well as a consensus on a process for integrating the patient perspective into clinical development will be of interest to many stakeholders. Geissler and colleagues have proposed a roadmap for early and continued patient engagement and collaboration throughout research and development of medicines (4). However, implementing this roadmap in daily practice can be challenging as numerous methods exist for assessing the patients' perspective and generating patient-based evidence but these are rarely used early in drug development (5). Large multi-stakeholder initiatives such as IMI-PREFER are ongoing to develop guidance on how patient preferences can be used to inform decision-making across the product lifecycle (6).

To further this debate regarding when and how to involve patients in a structured way in the drug development process, we have been exploring different methodologies and practical approaches. Based on our experiences, from an industry perspective, we postulate the following three hypotheses as a basis for implementing the patient engagement roadmap in a hands-on patient-centric drug development process:

*Our Primary Hypothesis:* Early and continued integration of the patient perspective into the development process for new health technologies, from phase 1 through to patient access to the products, will result in healthcare solutions with outcomes that best address patients' needs.

*Our Second Hypothesis:* A structured process to build patient-based evidence involving partnerships between patients and other key stakeholders (e.g., industry, regulators, payers, guideline developers, clinicians, healthcare systems) will improve alignment of development activities in accordance with the needs of patients.

*Our Third Hypothesis:* Quantitative patient preference research, built on robust qualitative insights, is necessary to strengthen the base for healthcare decision-making in the interests of patients.

To examine these hypotheses, this article reviews the current status of the discourse among the different stakeholders, illustrated with examples of how patient-based evidence can be generated, starting from desk research through to informing PRO strategies, clinical development plans, and product design or delivery features. This evidence should serve to transparently inform the early dialog with regulators, health technology assessment (HTA) bodies and payers.

## Methods and Processes for Patient-Based Evidence Generation

We describe examples from our recent research projects conducted in two disease areas, dry eye disease (DED) and non-alcoholic steatohepatitis (NASH), in which the patient experience and preference research has played or is playing a key role in the early product development decision process. The process and structure of this research is outlined in section Prioritization of development projects for generating new patient-based evidence and Creation of a structured process for gathering patient-based evidence and the methods for these studies are briefly described in sections Social media listening—example of a study in DED to Design of quantitative patient preference research.

### Prioritization of Development Projects for Generating New Patient-Based Evidence

We have established a standardized process for the generation of patient-based evidence, which can be applied in principle across a broad range of disease areas. Two important factors determine the extent of gathering early patient-based evidence: (1) pre-existing internal company knowledge and experience with patient needs in the target indications and (2) the knowledge, familiarity with and perception of patient needs of the stakeholders involved in appraising a new therapeutic innovation in that disease. If a lack of knowledge is detected in relation to the patient perspective, further investigation in the early development stages will likely be beneficial. If patient needs are sufficiently established and recognized by all stakeholders, additional investment into patient-based evidence at this early stage of development may be less of a priority.

### Creation of a Structured Process for Gathering Patient-Based Evidence

Robustness and completeness of the patient-relevant attributes and levels to be tested form the basis of a solid quantitative patient preference study. We have therefore designed a mixed methods research approach for patient-based evidence during early drug development including qualitative components and quantitative patient preference studies. The goal is to prioritize patient-relevant endpoints in the design of the clinical study program (7).

**Figure 1:**
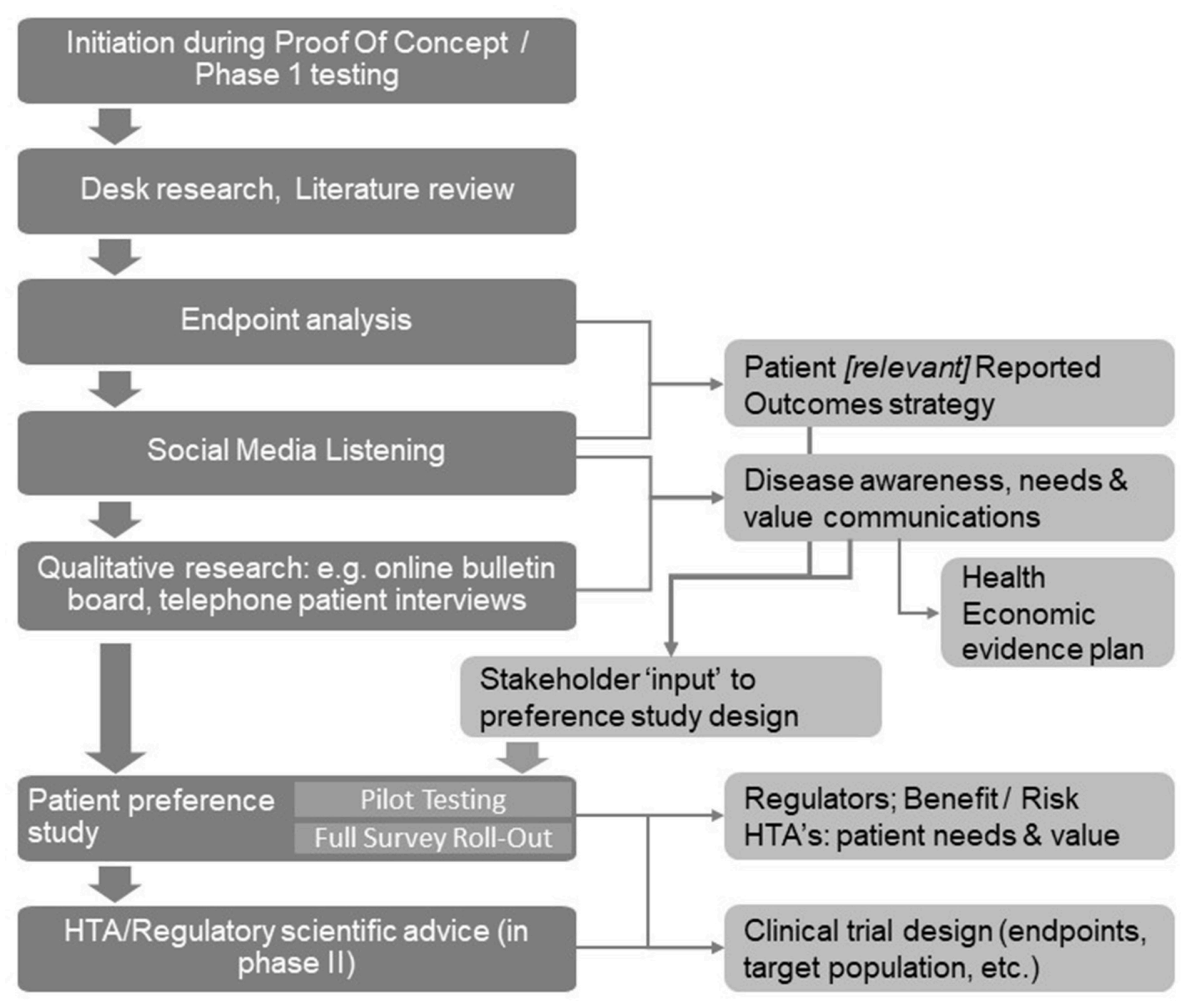
A structured process for patient insight gathering in early drug development [Adapted from Holtorf and Cook, (7)].

As shown in Figure 1, initial desk research and a targeted or systematic literature review helps to ensure familiarity with what is already known and published. Typically, this is followed by an observational analysis of patient conversations that are taking place online through open-access social media listening analysis relating to the indication of interest.

The learning's from the desk research and the social media analysis will then be corroborated and built upon further through interaction with patients (or caregivers) in the setting of qualitative research using online bulletin boards (OBBs).

OBBs are online closed community platforms that are used as a highly effective qualitative tool for research with patients (or caregivers). Invited participants anonymously engage in a moderated discussion over several days or weeks with other patients, comprehensively answering pre-defined questions, and responding and building on each other's posts.

The collective insights gathered through literature review, social media listening, and the qualitative research determine the design of the patient preference studies for the quantitative determination of the relative importance of the patient needs, and the trade-offs patients are willing to make. Before conducting the quantitative research, the survey questionnaire and language is tested and validated by in-depth telephone interviews with a representative number of patients in each of the target countries to ensure patient friendliness and face validity.

### Social Media Listening—Example of a Study in DED

For this review, the approach of social media listening analysis is exemplified by a study conducted in DED (8). In a retrospective study, DED-related posts were extracted from social media platforms using the *Salesforce Social Studio*^®^ tool (Salesforce, London, USA). Specific keywords related to DED along with search terms for symptoms, diagnosis and medications were used in the platform to extract the data set from English language posts. The extracted data were filtered and cleaned through algorithms included in the software and then manually by trained data analysts to remove duplicates, irrelevant and out of scope content. Using natural language processing, the posts were further indexed using patient- and disease-related lexicons to arrive at a sample set of 2,279 possible patient posts. These posts were further manually analyzed to identify the relevant posts from dry eye patients (*n* = 1,192) rather than from non-patients/caregivers (*n* = 1,087). Finally, relevant patient posts were individually reviewed and summarized to derive patient-specific qualitative and quantitative insights. The full methods are described in more detail elsewhere (9).

### OBBs—Example of Qualitative Patient Insight Generation in NASH

For this review, the methods of OBBs are described through the example of a study conducted in NASH (10). Patients with NASH in the UK (*n* = 8) and the USA (*n* = 8) were referred by their physicians to participate in two closed OBB forums. Over four consecutive days, the patients used aliases when going online at least twice per day and answered structured questions addressing (A) the medical pathway experienced by them; (B) their psychological and emotional attitude toward NASH; (C) the burden of NASH on daily living, finances, and employment; (D) the physician-patient interaction regarding NASH, including quality of relationship, emotions during consultations, and perceived physician attitude toward NASH; and (E) disease management approaches and patient expectations regarding potential new treatments for NASH. The OBB was asynchronous and a trained moderator facilitated the discussion, encouraging online group interaction. Participation in the OBB was entirely anonymized. Different qualitative research tools were combined to analyze the patient responses: content analysis to retrieve information relating to pre-defined questions, grounded analysis for identifying areas for further exploration, and discourse analysis to understand the context of patient responses and the meaning of the stated information to them. Follow-up questioning was possible within the group or individually. In the week following the OBB, the same moderator addressed any remaining open questions during a group teleconference with the OBB participants. Full details of the methods is described elsewhere (10).

### Design of Quantitative Patient Preference Research

Throughout the design and evaluation of the quantitative stated patient preference surveys, both medical experts and patient advisers/patient support group representatives were involved to ensure relevance, feasibility and face validity from patient and physician perspectives. During the design and optimization phase of the survey, routine telephone or face-to-face interviews with patients and patient group representatives were conducted to ensure user-friendliness and acceptance.

In the preference study for DED, a self-explicated conjoint design (11) was used and to study patient preferences in NASH, an adaptive choice-based conjoint (ACBC) analysis (12) was chosen.

#### Self-Explicated Conjoint Analysis for Testing Preferences of Patients With DED

An online survey was administered with 160 patients from the UK, USA, Australia and Germany (*n* = 40 per country). The survey construct was informed by the results of the preceding qualitative phase including a targeted literature review and a social media listening project as well as in-depth telephone interviews with selected patients (*n* = 3 each in Germany, UK, USA, and Australia).

The survey was accessible on a dedicated secure closed website. Patients were recruited through patient panels without direct involvement of physicians. To ensure recruitment of patients with moderate to severe DED, a patient screener assessed participants for the symptoms experienced [derived from the IDEEL dry eye PRO questionnaire (13)] and medications used.

Patient needs identified from the qualitative phase, categorized into 25 attributes, were included in the online questionnaire using self-explicated conjoint analysis to determine patient preferences across the four domains: treatment satisfaction (7 attributes), Symptom bother (8), Treatment administration (5) and Impact on daily life (5). Each attribute could be described in three to seven levels, which were scored by the participants relative to the best performance level score of 100.

For example, the attribute “treatment effectiveness on symptom relief of dry eyes” was represented by the following three levels: “treatments eliminate completely the symptoms of dry eye,” “treatments relieve most symptoms of dry eye,” and “no reduction in dry eye symptoms.” “Frequency of treatment use” was represented by seven levels: as needed, once daily, twice daily, three times a day, four times a day, once weekly, and once monthly.

Before weighting the four domains relative to each other, respondents first weighted the levels for each attribute and then the importance of attributes within a domain. To weight the levels within an attribute, the preferred option was given a score of 100 before the others were scored with 0 to 100 relative to the best. For example, if the attribute “eye pain” was being evaluated, they would consider “No eye pain” (score = 100) relative to “general eye pain” relative to “burning eyes” relative to “stinging eyes,” to assess the relative extent to which each eye pain manifestation is bothersome to them. Similarly, the relative importance of the attributes was determined within each domain.

Large text size, good color contrast combinations and the option to pause and return later made the survey more user-friendly for patients with DED. A survey experience questionnaire was administered at the end to assess user acceptance.

#### ACBC Analysis for Testing Preferences of Patients With NASH

A quantitative 30 min online survey was conducted with 153 patients with NASH from Canada (*n* = 36), Germany (*n* = 50), the UK (*n* = 17), and the USA (*n* = 50) to understand the impact of NASH on patients' lives, how they manage their health condition and their expectations for new NASH treatments.

The survey structure was informed by the results of a previous qualitative OBB research study and in-depth qualitative telephone interviews with 17 patients diagnosed with NASH from Canada, Germany and the UK. A steering committee involving a NASH patient, patient support group representatives, physicians specialized in liver diseases, and HTA advisers consulted on the design and conduct of the survey throughout the project.

Patients were recruited via physician referral if they had a diagnosis of NASH with suspected fibrosis level F2/F3. The online questionnaire included an ACBC exercise to quantify patient preferences for hypothetical product profiles (12). These were derived from a prior exercise in which patients ranked the product attributes that would be most important to them for a medical therapy for NASH. These attributes were then combined into six hypothetical product profiles and compared and prioritized pairwise by the patients in repeated choice exercises.

A survey experience questionnaire was administered at the end to test user acceptance.

The full methodological details are described in the publication on patient preferences in NASH (14).

## Results

### Social Media Listening Study in DED

The key themes of discussion in the 1,192 posts included in the analysis were *disease management* (1,393 mentions), *symptoms* (901), *causes* (409) including medical conditions, surgery or medication side effects, *diagnosis* (137), and *associated comorbidities* (187). The most common symptoms mentioned in the posts were *eye dryness, eye pain*, and *blurry vision*.

*Quality of Life* (QoL) was mentioned in 224 posts and described specifically in relation to the patients' daily routine including workplace difficulties, commute/driving and use of electronic devices, with high impact on their psychological wellbeing.

**Figure 2:**
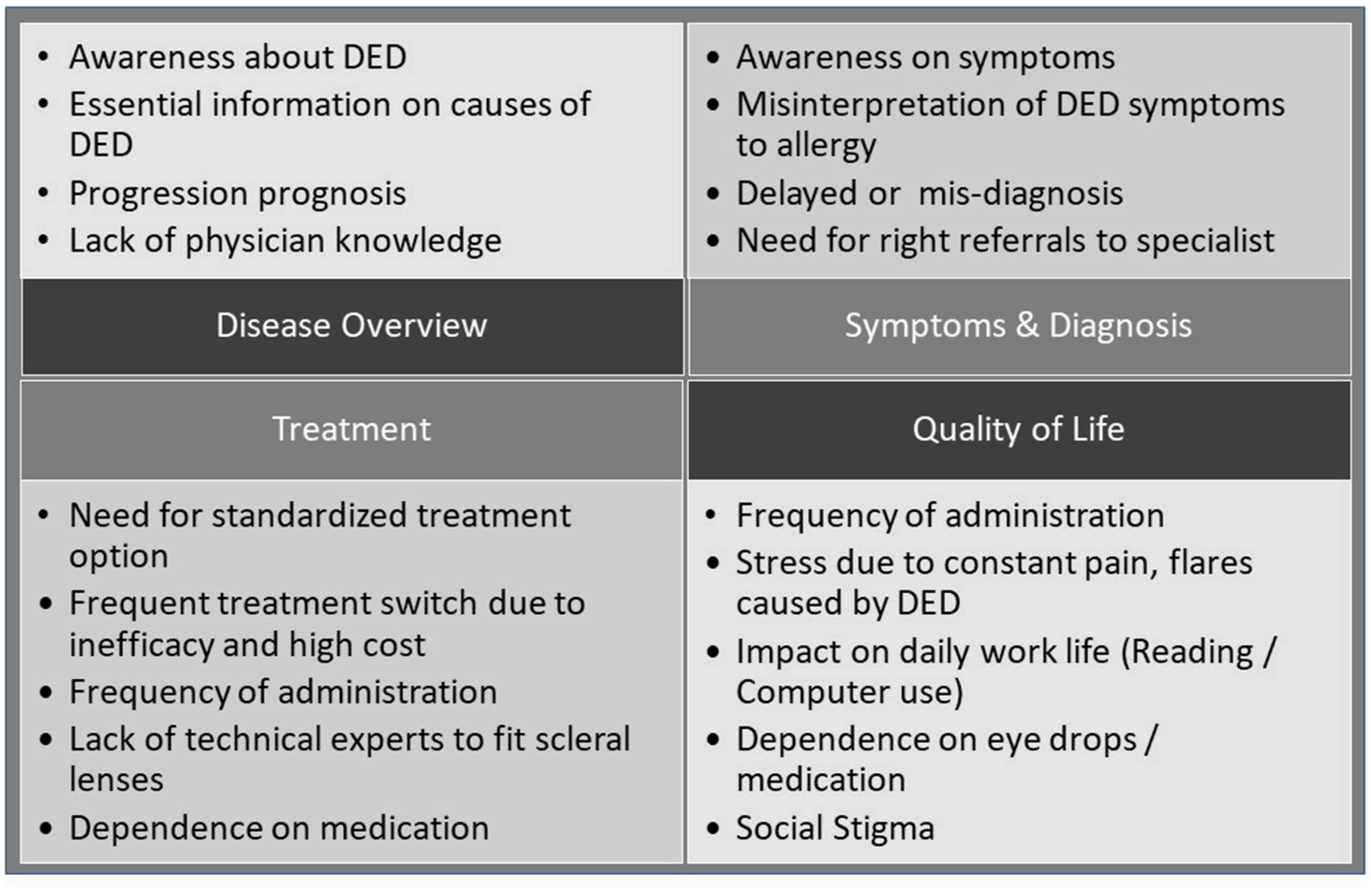
Patients' perspectives on DED: broad themes as resulting from the analysis of patient posts (15).

In terms of *unmet needs of the patients*, the statements identified in Figure 2 could be classified into four main categories, disease, symptoms and diagnosis, treatment, and QoL (8). Noticeable was a general lack of knowledge by patients about DED and its progression, difficulties in finding useful information on the disease, delayed diagnosis often with previous misdiagnosis due to low physician experience with DED, and a lack of DED-specific treatments beyond lubrication and symptomatic interventions.

### OBB Study in NASH

#### Patient Knowledge and Perceptions of NASH

Most of the OBB participants revealed a limited understanding of their disease and about half of them did not know the level of severity of their NASH. The participants did not seem to place a high importance on their liver condition: comorbidities such as obesity and type 2 diabetes were perceived to be more disconcerting than NASH. They feared, however, a decline in health, an uncertain future, and increasing need for medical interventions. Most patients felt that the disease was self-inflicted by unhealthy behaviors, and consequently, they expressed guilt and shame for their condition.

#### NASH Diagnosis, Monitoring and Management

For most patients, diagnosis had been incidental. The relatively low degree of confirmation by biopsy (50%) was explained by fears of the expected pain associated with biopsies. There was a general sense from patients of a low level of support from their physicians in relation to both disease education and the treatment or disease management options. The recommendation to lose weight was usually received but not effectively followed due to a lack of willpower and discipline to adhere to dietary and lifestyle changes as well as a lack of professional support in the endeavor. Only half of the patients were regularly monitored for NASH after diagnosis with frequencies varying from 3-monthly to yearly.

With respect to symptoms associated with NASH, fatigue or daytime tiredness were mentioned most frequently (mentioned by 11 of the 16 patients). Thereafter, patients referred to obesity (10/16), itching (9/16), and sleeping problems (9/16) and, less frequently, to weakness or lethargy (7/16), anxiety, depression, pain or flu-like symptoms (6/16 each).

Although the symptoms were mostly not perceived to impact their daily life, most patients reported that daily activities and work performance were compromised with progression of their liver disease.

#### Value Drivers of Future Treatments

Whereas some patients favored lifestyle changes, thus avoiding medical intervention with its potential side-effects, others mentioned that they have faith in drug treatment as they felt this could work faster and better than lifestyle modifications (which had failed them in the past). Some patients believed that by adopting a healthier lifestyle, they could reverse their condition, but they found it hard to stick to a weight loss or exercise regime or healthier eating habits.

Desired outcomes of future treatments were described as reversal of liver damage (within a year), improving quality of life (within a year), prevention of disease progression (within a year), reduction of symptoms such as fatigue and flu (within months), and as long-term outcomes, avoidance of organ transplant and prevention of disease progression (within 5–10 years).

### Quantitative Patient Preference Studies

**Figure 3:**
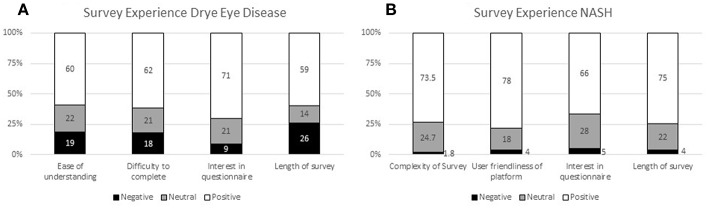
Survey experience in the preference questionnaires for **(A)** DED and **(B)** NASH.

The experiences of the respondents with the surveys in both patient preference studies (DED and NASH) are presented in Figure 3 (14). Patients required around 50 min on average to complete the DED survey using the self-explicated conjoint analysis (Figure 3A) and 30 min to complete the NASH ACBC survey (Figure 3B).

In the DED study, the patient survey experience was mostly positive with 71% of patients finding the survey interesting, 62% finding it easy to complete, and 60% easy to understand. However, whilst the majority of respondents (59%) found the length of the survey manageable, the results indicate that this survey length is at the upper limit of acceptability without causing respondent fatigue. Patients appreciated that they could pause/save and go back to the survey; 25% made use of this feature.

The survey experience of the NASH patients was rated similarly or even slightly better. The majority (73.5%) considered it easy to understand; 78% felt that the survey platform was easy to use, 66% indicated that the survey was interesting to them, and 75% of them felt that the time (30 min on average) was acceptable. Only a small minority gave negative ratings for these questions (1.8, 4, 5, and 4%, respectively).

**Figure 4:**
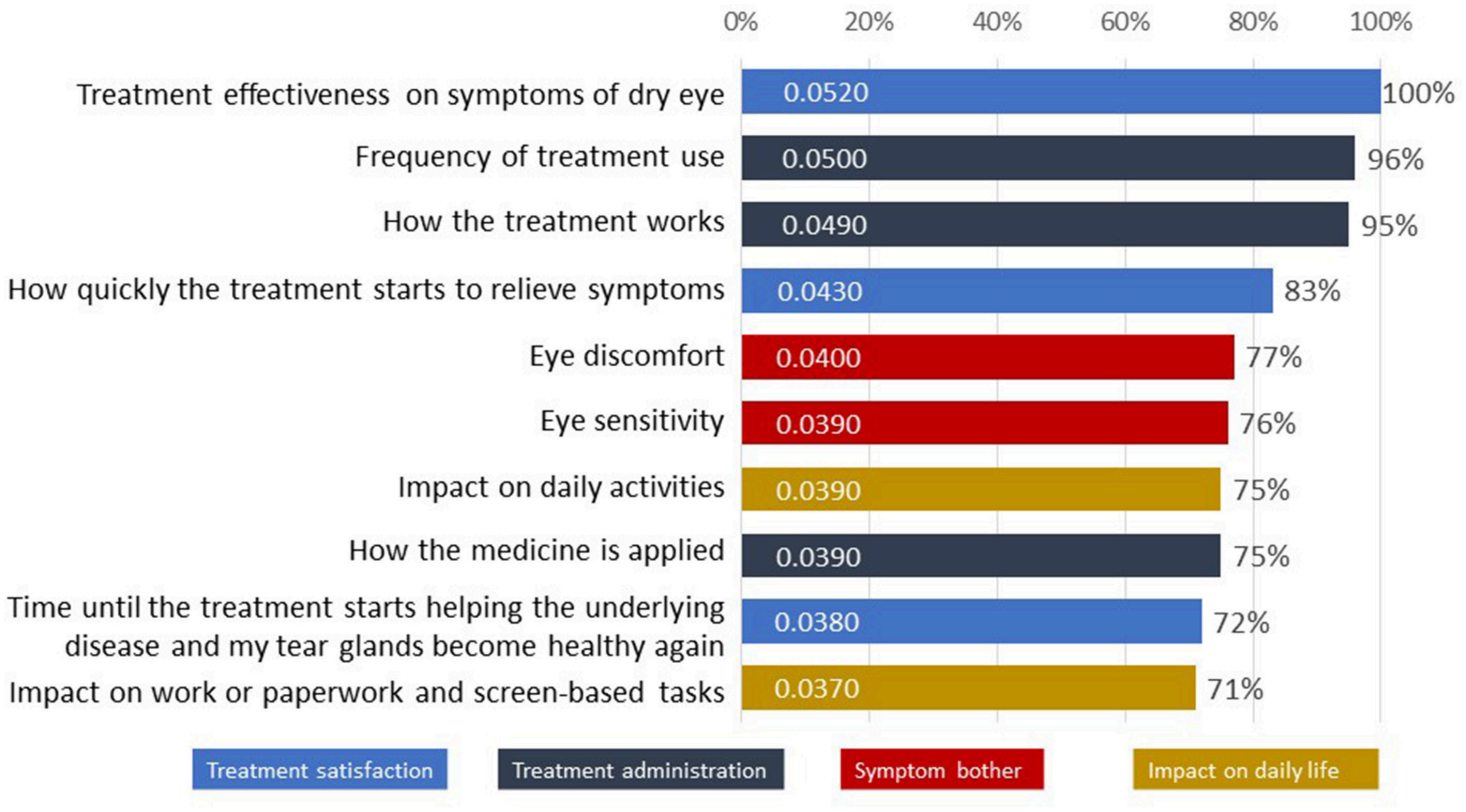
The 10 attributes most important to patients with dry eye disease as tested in the survey (16).

The ten most important attributes identified in the Patient Preference Study for DED across all four domains are listed in Figure 4 with “Treatment effectiveness on symptoms of dry eyes” (from domain “Treatment satisfaction,” 100%), “Frequency of treatment use” (“Treatment administration,” 96%) and “How the treatment works” (“Treatment administration,” 95%) as top priorities (16).

In total, 166 patients (36, 50, 30, 50 patients from Canada, Germany, UK and USA, respectively) participated in the Patient Preference Study for NASH. Of these, 53% received the diagnosis of NASH during routine visits or general health checkups and only 5% of patients were diagnosed because of specific suspicion of liver disease; all others were diagnosed as a secondary result when tested for other reasons, e.g., one of their comorbidities. Frequently reported comorbidities were obesity (71%) and diabetes or a pre-diabetic condition (53%).

Specific complaints of NASH patients relating to the disease experience were “lack of advice or support other than recommending weight loss,” “lack of counseling on diet,” “no referral to a dietician or nutritionist,” and “seen as secondary issue to the comorbidities.”

**Figure 5:**
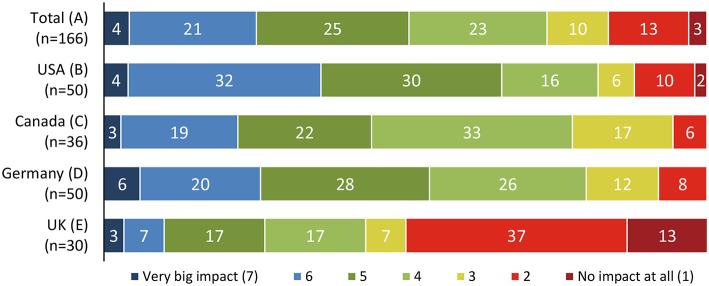
Perceived impact of NASH on the patients' overall quality of life (14).

Many patients did not note a direct impact on their health and well-being, with some variation across the different countries (Figure 5) (14).

**Figure 6:**
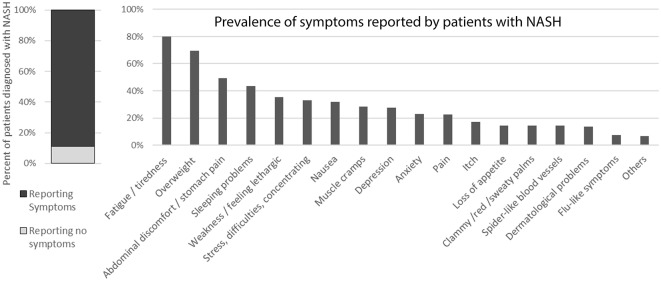
Symptoms reported by patients diagnosed with NASH. [The question was “Do you currently present any of these symptoms or conditions, regardless of what may have caused them (your liver condition or something else)?”].

In addition, the vast majority (approximately 89%) reported symptoms (Figure 6) but these mostly could not be pinpointed to the liver condition specifically as most of the patients also suffer from comorbidities.

**Figure 7:**
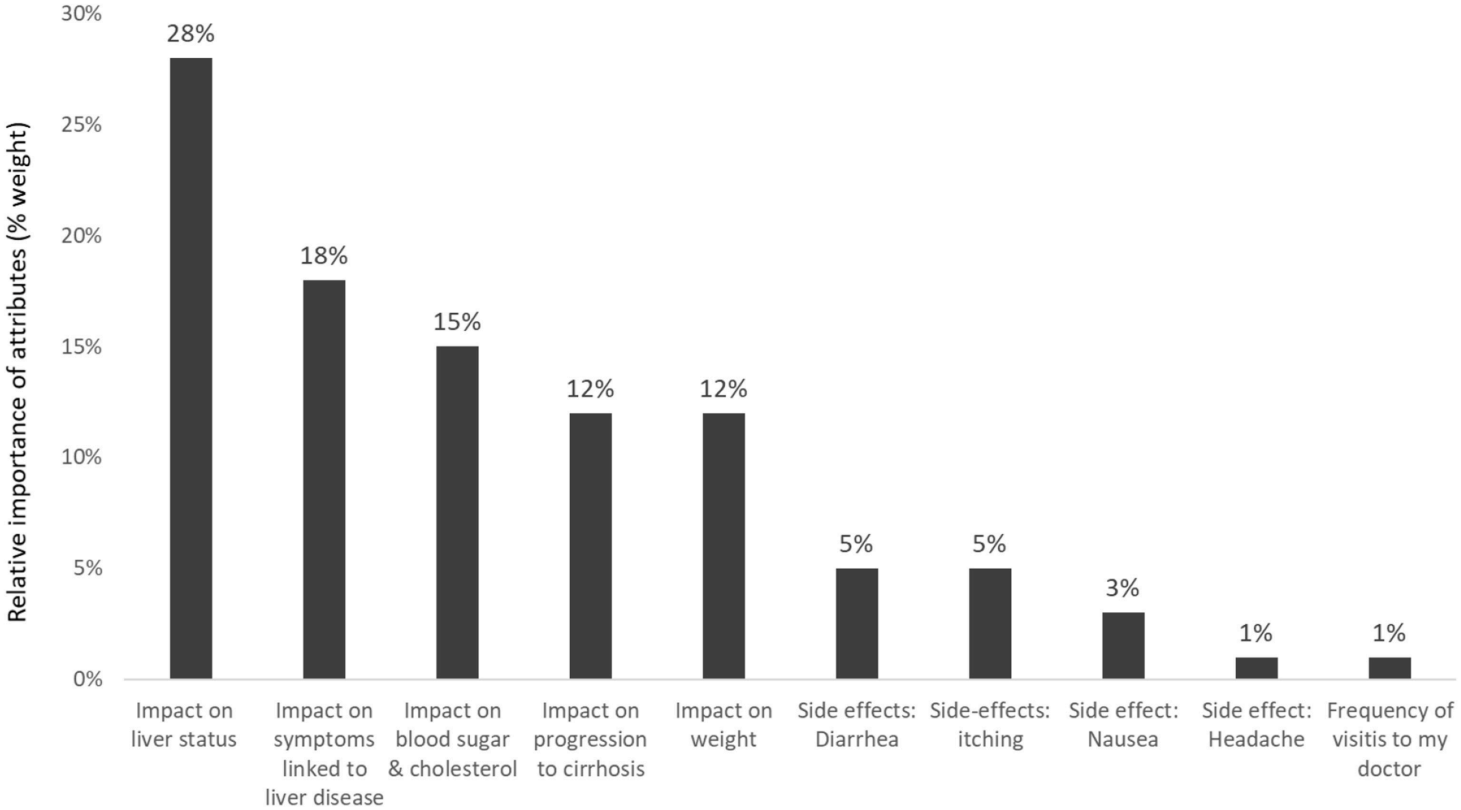
Relative importance of predefined attributes to patients with NASH (Pooled results of patients from USA, Canada, Germany, and the UK) (17).

For a new hypothetical treatment for NASH, the beneficial effect on liver status was rated the most important attribute (weight 28% of 100%) (Figure 7). This was followed by the reduction of liver-related symptoms (18%). The impact on weight, blood sugar and cholesterol, and delay of progression to cirrhosis were more important for patients than the four pre-defined hypothetical treatment-related side effects: diarrhea, nausea, itching, and headache (17).

**Figure 8:**
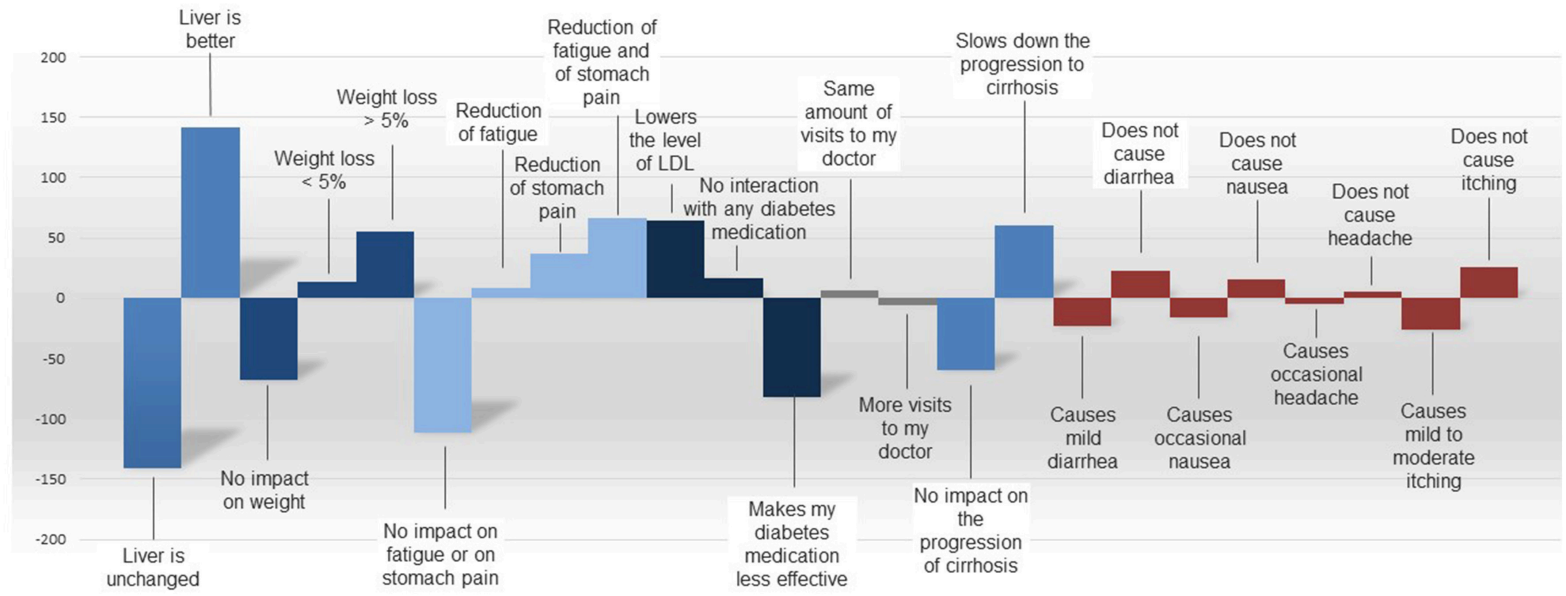
Utilities by attribute as derived from the comparison of product six hypothetical profiles of products for NASH.

Liver efficacy was also the most important predictor for product choice by NASH patients in the comparison of hypothetical product profiles. Other values, such as improved side-effect profile, direct impact on weight loss, slowing down disease progression, or the same number of practitioner/specialist visits are supportive elements for product choice. The utility values between the tested attributes as derived from the comparison of product profiles are depicted in Figure 8.

In trade-off decisions by NASH patients, product-related side effects such as diarrhea, nausea, headache, and itching were generally perceived as less important than expected benefits such as reduction of fatigue or stomach pain, or slowed progression of cirrhosis, across all countries.

## Discussion

By presenting early research on patient preferences in DED and NASH we aim to show real-life case studies supporting our three hypotheses as formulated in the introduction.

In both the DED and NASH examples, the patient perspective has been obtained in an early stage of drug development (phase 1–2) and was gradually built up in a structured process starting with the collection of purely observational (literature review/social media listening) and later interactively generated (OBBs/telephone interviews) qualitative evidence, which then fed into the design of quantitative patient preference studies. The intention was to develop a strong evidence base for the range of patient experiences, priorities, and reactions to potential new therapies. This patient-based evidence can be used to inform early discussions with various stakeholders and, ultimately, to ensure that the evidence generated in the phase 3 clinical studies reflects patient-relevant outcomes.

Such research meets the increasing requests that the evidence the pharmaceutical industry brings to the table for its new products not only includes efficacy and safety data but also endpoints that are relevant to the target patients and that the product will deliver “patient value” (5, 18–20). Currently, patient-based evidence is still not reported sufficiently in pharmaceutical company submission dossiers, as reported by the Canadian HTA agency (CADTH) when they reviewed dossiers submitted between December 2012 and June 2014 (21). Examples of HTAs formally assessing patient-based evidence or product labels incorporating patient data and patient value elements are still, sadly, few and far between (22, 23). However, to be fully involved in decisions related to their health is considered important by patients. From their perspective, patient preference information could lead to better decisions and higher acceptance of therapies by patients, as indicated by the example of rheumatoid arthritis (24).

Therefore, a greater involvement of patients during the design phase and use of quantitative patient preference data and patient expertise may improve the effectiveness of product development. To examine our three hypotheses in more detail, we will dissect and discuss them one by one.

### Hypothesis 1: Patient Input Should Be Elicited Early in the Drug Development Process

Our primary hypothesis proposes that early and continued integration of the patient perspective into the development process for new health technologies, from phase 1 through to patient access to the products, will result in healthcare solutions with outcomes that best address patients' needs.

Patient experiences of the disease and therapy including efficacy and side effects, drivers of patients' choice of treatment, and patients' definitions of meaningful therapeutic improvement are all factors that are playing an increasingly important role in the assessment of new therapies. Many agencies involve individual patients or patient representatives to inform the regulatory or HTA process (*patient input, patient involvement*) (25). In this function, patients can contribute their *experience* of living with the disease and thereby support the interpretation of the clinical evidence with impact on the decision to make new interventions available (18, 25, 26). Likewise, patients can contribute to the design of clinical trials by being *collaborative research partners* for the research team (27).

To increase the representativeness and complement *patient experience* testimonies for regulatory and HTA decision-making however, more systematic patient research is needed (23). Methods summarized under the synonym of “*patient-based evidence”* aim to represent the *patient perspective(s)*, albeit at a higher level of consolidation, with broader representativeness, and generalizability (28). *Patient-based evidence* can consolidate patients' experiences, perspectives, perceptions, needs, preferences or attitudes about their care and health (29). The concept of patient-based evidence typically comprises a level of co-production and patient participation but also allows for patients (or patient organizations) to collect such evidence by themselves (30).

Our examples of generating patient-based evidence in early development in NASH and DED show how industry can respond to the demand for greater patient-centeredness. By involving patients and generating patient-based evidence throughout research and development, product design and evidence generation is more likely to concentrate on the health outcomes that are most relevant to them (3, 6). To enable optimization of patient-oriented product development, the research to define the needs of patients and relative importance of certain attributes must precede the design of the phase 3 clinical development program (31, 32). Time is needed for creation and validation of new PRO instruments that may be needed to address the patient-relevant endpoints, so that they can be used in pivotal phase 3 trials. Only if patient insight and preference studies are initiated in phase I/early phase 2, can they inform and complement the PRO strategy in a timely manner.

As has been pointed out by others, considerable financial value can be generated through early integration of the patient perspective, due to more targeted and faster development, improved clinical trial realization, and reduced uncertainty in the technology assessment phase (3). For *the trial design*, early qualitative and quantitative patient-based evidence research can add *essential value* in several ways: (1) informing the choice of outcomes measures including the PRO measures and digital evidence collection; (2) minimizing the burden of the study protocols to the patients or their caregivers by considering the patient characteristics in the trial protocol; and (3) informing the development of educational material around the clinical trial.

In fact, there are a range of ongoing initiatives that aim to develop and apply agreed standardized sets of outcomes, also known as “core outcome sets,” which “represent the minimum that should be measured and reported in all clinical trials of a specific condition” (33). The expectation is that the core outcome sets will be collected and reported consistently in any clinical or outcomes research in a specific disease, making it easier for the results of trials to be compared, contrasted and combined. The International Consortium of Health Outcomes Measurement (ICHOM) has defined its mission as to “unlock the potential of value-based healthcare by defining global Standard Sets of outcome measures that matter most to patients and driving adoption and reporting of these measures worldwide to create better value for all stakeholders” (34). As can be seen, the emphasis on *patient-relevant* outcomes supports our proposal for rigorous patient preference research in each disease to assemble a solid evidence base for what “matters most to patients.” In the beginning of 2019, ICHOM lists 27 completed core outcomes sets and an additional 9 that are in development (34). Likewise, the CMTP/Green Park Initiative in the USA is developing core outcomes sets and “CoreNASH” is one of the ongoing projects (35). The mission of this initiative is to “make healthcare more effective and affordable by improving the quality, relevance, and efficiency of clinical research.”

In our example of DED, identification of patient preferences before starting the clinical studies revealed the importance of measuring both the effect on the underlying disease, as well as measuring the impact on symptoms such as discomfort, sensitivity, pain, and fatigue of the eye [symptom bother (13)].

If the numbers of patients in the preference study allow, one can also purposely investigate potential differences in preferences among subpopulations. In the example of the NASH preference study (14), we could observe geographical/cultural differences in the preferences derived that might be explored further in subsequent studies. Similarly, one could investigate potential differences in preferences of younger vs. older patients, by gender, or differences related to different comorbidities, stages of the disease or symptoms experienced. Such information could be important for healthcare providers in making the best treatment decisions for the different patients, taking account of their individual needs, preferences and expectations at the point-of-care (36).

Across both studies in DED and NASH, the patients seemed to assign more importance to alleviating “noticeable” effects (symptom relief) than to effects on the underlying disease. This differs from the priorities assigned by hepatologists and internists treating NASH patients (unpublished, manuscript in preparation). Similar differences in preferences for treatment outcomes have also been observed in rheumatoid arthritis (37, 38). In addition to improving the clinical trial study protocols, such observations can also help to improve the communication between doctors and patients and facilitate shared decision making and evidence-based medicine in clinical practice (39).

Beyond shaping *product design features*, early preference studies (at a time-point when changes to the clinical program may still be possible) could also guide manufacturers in the *co-development of services* or in *portfolio management* decisions. For example, the DED patient preference findings indicate that patients would like future products to fulfill a number of specific requirements including (a) provide symptomatic relief and lubrication, (b) be fast acting, and (c) be applied when needed rather than in fixed dosing frequencies (16). The NASH study highlighted in particular the need for patient education on the disease and its likely progression, as well as an improvement in the patient–physician consultation and information exchange around NASH (16).

Nevertheless, there are a number of potential downsides of starting such preference research in the early phases of product development. For the manufacturer, this is an additional up-front investment that may not bear fruits, should the asset in question not advance into full development for whatever reason. Moreover, such involvement requires investment and engagement from patients, and may give rise to hope and unrealistic expectations regarding availability of new therapies. However, much of the evidence generated in these early research and development phases will be product-independent and, if published, could guide others in establishing core outcomes measures (33) and in the development of product and services. A further limitation of starting early with preferences research, is that patient preferences may change over time as new therapies become available that address some of the needs, or if new cultural or experiential aspects impact on patient thinking.

### Hypothesis 2: Stakeholder Alignment Should Be Built Around Requirements for Patient-Based Evidence

Our second hypothesis is that a structured process to build patient-based evidence involving partnerships between patients and other key stakeholders (e.g., industry, regulators, HTA/payers, guideline developers, clinicians, healthcare systems) will improve alignment of development activities in accordance with the needs of patients.

The two important components of this hypothesis are that a structured process for generating patient-based evidence needs to be established, and that a consensus on this is reached among the key stakeholders in the advancement of products and services in healthcare.

#### The Importance of Structured Research for Patient-Based Evidence

Although a transparent process forms the basis for continuous learning and improvement, it does not mean that each step of the process is relevant, and thus mandatory, for each new asset in development and in each disease situation. Rather, the process should serve as a framework, whereby the importance of generating new patient-based evidence needs to be considered by the manufacturer together with input from a multi-stakeholder advisory board (including patients) throughout development. This notion parallels the recognition of HTA agencies, that it is “imperative that we clarify when patient involvement is likely to add value” (18). From the HTA perspective, the generation of patient-based evidence in early product development is encouraged to reduce the burden on patients and patient groups later in the product life cycle (18).

#### Integrating Different Stakeholder Views and Expectations to Advance Patient Centricity

Part two of our second hypothesis adds that not only a structured process but also the partnership between stakeholders is essential to improve the current research, development, and decision-making, to achieve a more patient-relevant approach and consequently, better patient outcomes. Although there are numerous barriers and rigorous guidelines that need to be adhered to concerning patient engagements and collaborations with industry (and other stakeholders), there are also good examples emerging of effective co-production between the different stakeholders (40). In some disease areas, such as rheumatoid arthritis and myeloma, patient–research partnerships are well established and their value for improving research and the decision base by HTA and regulatory bodies is becoming well recognized (2, 4, 27, 41, 42). Prominent examples of co-producing patient preference research are that Myeloma UK and Melanoma Europe played leading roles in respective studies for preferences of different stakeholders and acceptability of benefit–risk trade-offs (36, 43).

The Food and Drug Administration (FDA) in the USA conducted 24 disease-specific meetings recently under the auspices of the Patient-Focused Drug Development framework (44, 45) and a framework for incorporating patient preference evidence in the *regulatory assessment* regarding benefits and risks of medical technologies was published by Ho et al. (46). In fact, companies now need to include information on patient engagement in a dedicated section of the submission template in all submissions to the FDA (47). The European Medicines Agency (EMA) has engaged in patient preference studies in advanced myeloma (2) and multiple sclerosis (48) to better understand patients' willingness to accept risks as a trade-off for efficacy, and is systematically including patient representatives in the majority of their advisory and decision-making committees.

Although some *HTA bodies* encourage elicitation of patient preferences early in the development lifecycle (18), there is still a lack of clarity or guidance as to which qualitative or quantitative methods should be applied to conduct preference studies in a way that they optimally inform the HTA decision-making process (23). From pilot studies using discrete choice experiments and analytical hierarchy methods, Germany's Institute of Quality and Efficiency in Healthcare (IQWiG) concluded that both methodologies have merit (49, 50). Based on their experiences, IQWiG recommends the analytical hierarchy process to provide suitable endpoints for clinical trials in indications such as depression and cancer whilst they suggest the use of discrete choice experiments in health economic evaluations to identify, weight, and prioritize multiple patient-relevant outcomes (51, 52). The UK Institute for Health and Care Excellence (NICE) is considering the use of discrete choice experiments in the context of their economic evaluations, but it is still being debated whether and how this should be done (23). In the guidelines of the Australian Pharmaceutical Benefits Advisory Committee (PBAC), discrete choice experiments are mentioned as a potential method to elicit preferences in the economic evaluation (53). Building economic cost-effectiveness models around utility values derived from patient preference studies (54, 55), is an interesting area for future research, which is also being investigated by the IMI-PREFER project (56).

The Scottish Medicines Consortium (SMC) is now mandating a Summary of Information for Patient Groups (Section 8 of the “New Product Assessment Form” from July 2018) as an obligatory part of the new product submissions by the manufacturer (57). Consequently, manufacturers must summarize each new product submission in language that is intelligible for lay readers; those companies who have engaged early with patients and built robust patient-based evidence to inform their development strategies, are likely to be well positioned to meet these new demands.

**Figure 9:**
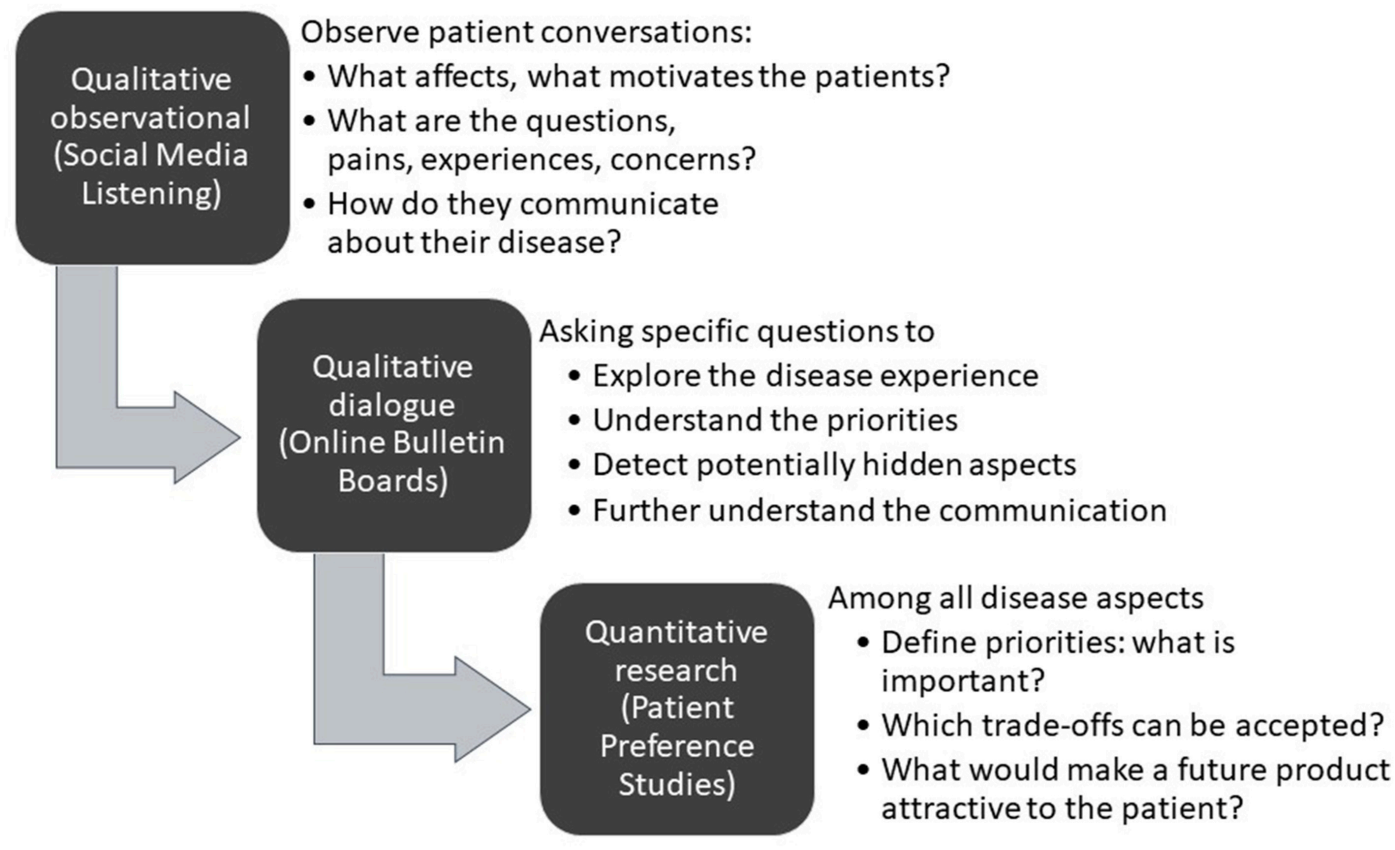
Sequence of questions to be answered throughout the quantitative and qualitative phases of patient insight and preference studies.

Many of the changes in the processes described above have been triggered by increasingly strong *patient organizations* (4, 58, 59). Patient experts can help as partners in study teams and oversight boards to achieve the objectives of the qualitative and quantitative research, as outlined in Figure 9, by ensuring that research questions are framed in a patient-relevant way, that study designs are feasible and relevant from the patients' perspective, and that the evaluation, results and conclusions have a face validity with patients. Engaged patient organizations therefore advocate: “Nothing about me without me” (60).

It should be noted however, that collaboration between patient groups and pharma companies on patient preference studies may raise some concern. Some feel this relationship could lead to bias and may compromise the neutrality or independence of the patient organization, especially if the manufacturer is also providing funding to them (61, 62). Such concerns may be less pronounced for research in the pre-competitive space or in early and product-independent research phases (40). A structured process, transparency, publication of the studies, and clear guidelines governing such collaborations will contribute to appease these concerns and provide a framework for future collaborative interactions (42, 63, 64). As in translational research, researchers and “users” need to interact early to optimize the utility of the research results (65).

Despite the growing emphasis on patient-centeredness in healthcare and also in HTA assessments, there is to date few examples of where patient preferences have informed the decision-making process (22). Yet, patient preferences identified and quantified during early development can feed into early dialog or scientific advice consultations between pharmaceutical manufacturers and regulators/HTA agencies (https://www.eunethta.eu/early-dialogues/), typically conducted in advance of finalizing phase 3 design. The first example of an HTA body offering scientific advice on the design of an early patient preference study has recently been communicated (66). Patient-based evidence and preference data are of high evidentiary and economic value if they help to avoid protocol amendments, improve enrollment, adherence, and retention in clinical trials (3).

While the concept of early dialog has gained acceptance in Europe (67, 68) in the form of the Early Dialogue and Joint or Parallel Scientific Advice, such a platform for alignment with payers is lacking in the US. The establishment of a US multi-stakeholder early dialog platform could greatly enhance the development and availability of technologies that are better tailored to the needs of patients. Whilst the FDA have been progressive in embracing the patient perspective and fostering new approaches to patient engagement, US payers still have headroom to incorporate the interests, needs and preferences of patients in their formulary decision-making. The addition of an early alignment format with payers could move the discussion from cost to aligning on the value for all stakeholders, including payers and patients. Such a platform would also support the move toward patient-centric healthcare and value frameworks as proposed by the Institute for Clinical and Economic Review (ICER) (69) and the National Health Council (70).

Integrating stakeholder views may not be universally possible: not all stakeholders may agree to become part of the process and, in some countries, it may be more difficult to establish a platform for early advice many years before the product is to be assessed. As can be seen from the horizon-scanning activities of HTA agencies, the time horizon of many HTA agencies activities often starts later in the development lifecycle, typically in the 2–3 years before submission. In addition, some healthcare decision processes may not prioritize patient centricity or patient-relevant outcomes in the same way as now observed in Europe or the North America.

However, we believe that engaging multiple stakeholders through early dialog/scientific advice platforms is a real opportunity to obtain alignment among stakeholders. Where possible, early research on patient preferences conducted in a structured and transparent process and aligning stakeholders around the specific need for and benefit of new therapeutic options will lead to more patient-focused drug development, reduce uncertainty, and will benefit all: physicians, patients, health policy makers, and industry.

Finally, it should be noted that the patients' perspective is only one of several inputs to decision-making and sometimes, it may not be aligned with other priorities or perspectives such as the societal perspective. To understand such potential points of conflict and to manage expectations from all sides is another important motivation for the stakeholders to interact early.

### Hypothesis 3: Patient Preference Research Should Be a Strong Pillar of Future Healthcare Decision-Making

In our third hypothesis, we suggest that quantitative patient preference research built on robust qualitative insights is necessary to strengthen the base for healthcare decision-making in the interests of patients.

When deciding on adopting or reimbursing new healthcare interventions, decision-makers want to understand—based on scientific evidence—who is the patient or what is the problem (target *patient population*), the exact nature of the intervention or exposure (*indication*, label), the current available therapy (*comparator*), and what improvements have been shown relative to the current therapy (*outcomes*) (71). This concept is known by the acronym PICO (patient/problem—indication—comparator—outcomes).

Building the evidence stepwise—from literature reviews and social media analysis to in-depth quantitative research—provides consistency and face validity and the resulting information will help to answer the PICO questions: the research will improve our knowledge on the patients (P) and their problems, what their life with the indication (I) is like, which current therapies they use and experience (comparator C), and which outcomes (O) are most relevant to them. The evidence on each of these levels can be used for downstream decision-making throughout development and can impact subsequent discussions with regulators, HTAs, and payers.

The patient perspective will not become the only driver for evidence generation throughout clinical development and evaluation. However, it should complement the clinical and the economic perspectives and, the stronger the evidence, the better it will support alignment across the different stakeholders.

#### Why Conduct Qualitative Research in Optimizing the Design of Quantitative Preference Studies?

Patient interactions on social media platforms are relatively quick to analyze and can yield insightful patient- and disease-relevant information (72) without imposing any research burden on patients. In addition to revealing the themes important to patients suffering from a specific disease or their caregivers, “social media analysis” is also a good approach to understand the terminology typically used by patients and caregivers when talking about an illness in an unconstrained and unprompted manner. The FDA has published guidance around using this type of data for pharmaceutical purposes, which was derived from several stakeholder workshops in 2018 (73, 74). Our Social Media Listening study in DED illustrates how important gaps in health care are communicated among the patients, which can be related to the disease itself (a perceived “lack of knowledge and awareness by doctors”), to the diagnosis (misinterpretation of symptoms, delayed diagnosis or wrong diagnosis), or to the therapy (lack of treatment options, high variability and frequent switching, administration frequency, access to therapy). Information extracted from observing thousands of patients helps in understanding the patient language and the issues at the forefront of their minds and to structure subsequent and more in-depth qualitative research. A shortcoming of this purely observational social media research is that clinical and socio-demographic information is often limited (severity of disease, detailed diagnosis, comorbidities, gender, education, etc.) as much of this information is not revealed in the patients' posts. In addition, the value of Social Media Listening is limited to those aspects of the disease that are “notable” to patients and may not reveal much information in asymptomatic diseases. We saw this in our own research, when we observed the online communication of NASH patients with milder stages of the disease in social media platforms (data not published), which yielded limited insights from the patients' unprompted online conversations.

In contrast, OBB research can help to challenge or confirm insights from previous research or to gather more focused information on specific domains or attributes that may be relevant to the patients. The OBB method has the benefit of running over several days or weeks, hence it allows clarifying or follow-up questions to be asked, or one person's comment to be built on through encouraging others to express their views.

With NASH, the interactive format of a moderated online discussion forum helped us to explore patient experiences regarding patient-perceived impact of a disease state such as NASH and the shortcomings of current disease management strategies. The structure of an OBB and the specific questions are formed from the knowledge gained from the previous research steps. As the conversation evolves over several days, OBBs provide the opportunity to interrogate new themes that arise, ask for clarification, or explore commonality and differences among the participating patients. A large volume of patient information is generated, in their own words and terminology, by asking focused questions. OBBs deliver the benefit of focus group discussions to be able to probe deeper in areas of interest, without certain individuals dominating the conversation. As the participants are anonymous, OBBs can help in uncovering patient experiences that might not be revealed in focus groups or telephone interviews, particularly on more sensitive, embarrassing, or emotional issues that people may have difficulty to openly talk about.

In some cases, it may be difficult for patients to truly express their needs for a new therapy; patients who do not appreciate the progressive nature of a disease may not spontaneously see the benefit of new treatments to address this issue. With NASH patients, due to a lack of disease understanding and the presence of comorbidities, patients had difficulty in relating symptoms with their liver disease. Appropriate educational support relating to the disease and its progressive nature may reinforce the patient value perception (75, 76).

Other examples in which the methods would have to be tailored to fulfill the special contextual requirements could be pediatric patients, where parents might have to be involved in the research, or in rare diseases, where low patient numbers may prohibit methods that depend on a high number of responses.

#### Methodological Considerations for Early Patient Preference Studies

At an early stage in the product lifecycle, the research questions and the required output from preference studies may well differ from those in later stages of development. Therefore, the choice of preference research methodology may also differ from the methods typically utilized for preference studies conducted closer to the time of submission to authorities (31, 77). For example, in a new, previously unexplored disease one may be confronted with multiple attributes of interest that may exceed the reasonable number to test in a discrete choice experimental design (typically up to ~7 attributes). A second consideration is that the costs associated with large sample sizes, as required in some study designs, may be prohibitive when dealing with early development assets.

When searching in mid-2018 for literature examples of patient preference study methodologies conducted during early development, we identified only 9 that were done in the early research and discovery phase (4 in oncology, 2 in respiratory, 2 in immunology, and a 1 in rare disease), and only 12 in the clinical development phase (1 in oncology, 2 in cardio-metabolic, 1 in hematology, 1 in respiratory, 4 in immunology, 1 in ophthalmology, and 2 in orphan diseases). All others related to products in the market authorization or HTA assessment phase (9 studies) or to products in-market (55 studies). Most studies in the early development phase used mixed methods (71% qualitative and 29% quantitative) and aimed at understanding burden of disease, health-related quality of life (HRQoL) impact of the disease for PRO development and validation, diagnosis management and benefit/risk trade-off. The quantitative stated preference methods most commonly used were discrete choice experiments; other methods used were best-worst scaling studies (BWS), analytical hierarchy processes, or other conjoint analyses. No published guidance on preference studies was retrieved for early development phases, in contrast to the market authorization phase or the HTA phase (44, 53, 77–84). Possibly, pharmaceutical companies commission preference studies as part of their market research, other commercial activities, or internal decision-making without publishing the results in peer-reviewed journals. Any unpublished research will not add to the body of evidence in the long term. If designed with clear objectives, validated methods, solid analysis, and published in peer-reviewed journals, the patient preference data and evidence not only support an informed discussion concerning one specific product but may contribute to fostering the development of therapeutic alternatives for all patients with that disease by highlighting patient needs, preferences for product attributes, or how to improve disease management and its associated symptoms, and by informing core outcomes sets for the respective disease areas (18).

The jury is still out on which methods lend themselves optimally to which research questions and at which stage of the development lifecycle (85, 86)—indeed, the PREFER consortium funded by the Innovative Medicines Initiative (IMI), seeks to address exactly these questions. Scientific experimentation with different approaches is therefore an important contribution to this process of discovery and learning (56).

Here, we have discussed two examples, the self-explicated conjoint analysis (in DED) and the ACBC analysis (in NASH). The self-explicated methodology (11) was chosen because of its ability to handle a large number of attributes as identified in DED, while still providing low cognitive strain on the respondents. A previous methodological comparison confirmed that self-explicated approaches, due to the straightforward research design, data collection, and data analysis, require less time and investment than traditional methods of conjoint analysis^1^, which is advantageous in early development phases (11). However, this study design is not suitable to identify benefit–risk trade-offs and therefore would be less suitable for regulatory decision making at the product submission stage.

The strength of the ACBC methodology is the dyadic design, whereby first the patients' relative priorities for attributes for NASH treatments were determined and then hypothetical product designs and variations were composed from these attributes, from which the patients selected their preferred options (12). This design helped to keep the survey relatively easy for the patients and allowed for completion within 30 min, despite the high complexity of NASH in terms of both symptoms and impact on HRQoL. In addition, we were able to investigate product design features and compare hypothetical future product profiles, as well as to analyze which trade-offs patients may be willing to make.

**Figure 10:**
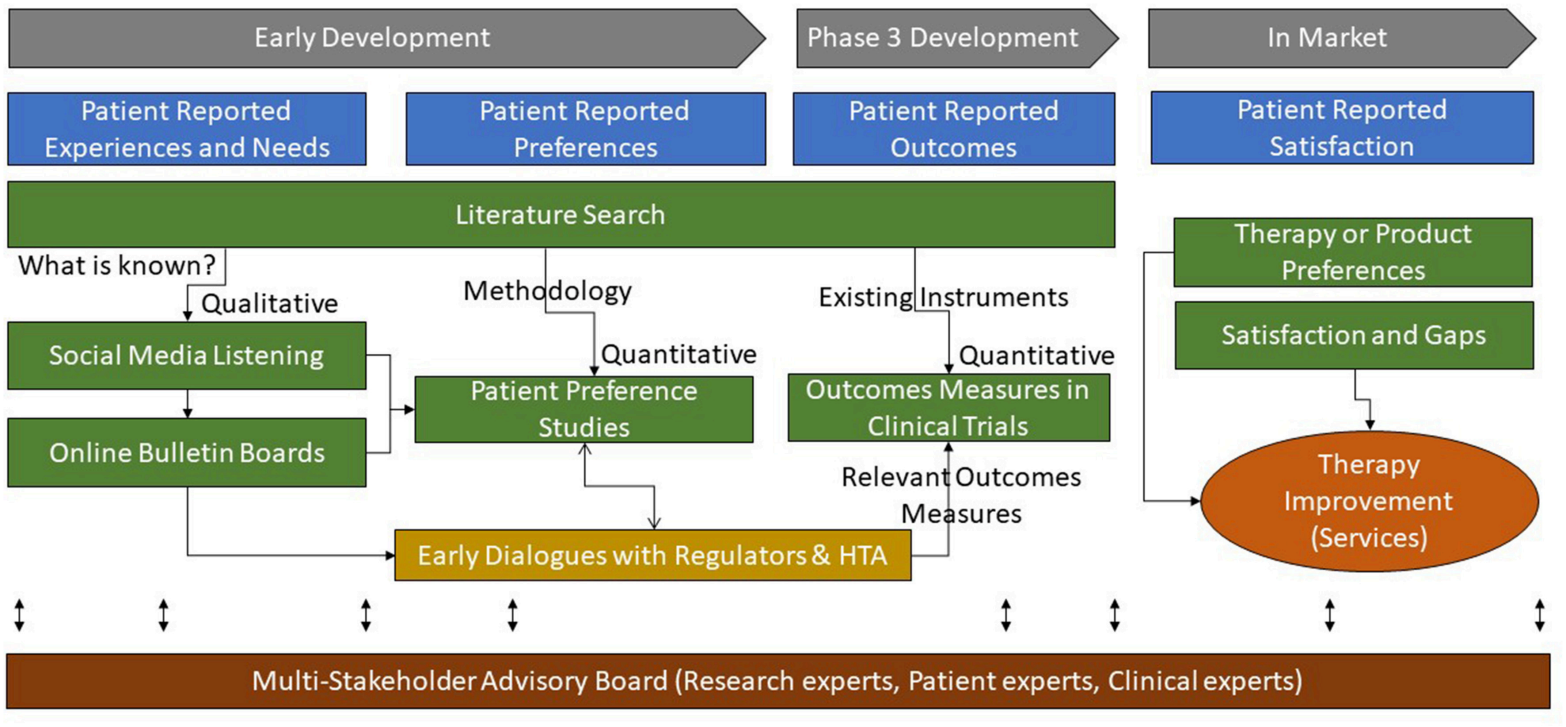
Multi-method approaches to research for patient-based evidence throughout the product life-cycle.

The primary incentive for industry to invest in patient preference research early in the product development cycle is to optimize how a future product can best address the needs of the patients as well as meet the requirement of healthcare systems, to improve overall health outcomes. Without adopting this concept throughout the entire product development process (see Figure 10), industry will run the risk of developing products inefficiently, with a regulatory and economic focus only, without addressing what is of greatest importance to the end user, the patient (3).

Klose et al have classified “Patient reports on healthcare” into four categories: preferences, outcomes, experiences, and satisfaction (31). In our proposed process, as delineated in Figure 10, patient experiences and satisfaction (or dissatisfaction) with the current level of care should be understood early to inform the design of preference studies. The preference studies should prioritize which outcomes measures best reflect the value of new products from the patients' perspectives and should therefore be integrated into the clinical development plan. In addition, this patient-based evidence can guide researchers and decision-makers in agreeing on the relevant outcomes measures (e.g., core outcomes sets), design, and context in the developmental clinical trials.

## Conclusions

Drug development with a focus on patient need is evolving to become an essential part of the pharmaceutical research and development process, both to better meet the demands of regulatory and reimbursement authorities, as well as the needs and expectations of the patients. Companies who embrace the involvement of patients in early product development, prior to beginning pivotal clinical trials, are most likely to ensure a fit of their products to the real needs of the patients and provide the therapeutic outcomes they are looking for. The first examples are emerging where such patient-based evidence and preference data are informing early interactions between developers of new medicines and HTA or reimbursement bodies. A more structured and systematic early alignment across different stakeholders and decision-makers could further improve the efficiency and relevance of drug development for all parties involved. First of all, it is necessary to have agreement that the evidence generated throughout the clinical development process should demonstrate improved patient-relevant outcomes. Subsequently, alignment of all stakeholders on structured and evidence-driven methodologies and processes to develop comprehensive and relevant preference research should help to improve patient-relevant outcomes starting from early development through to product prescription and utilization. A transparent process for generating such evidence is needed to measure the effect for patients, improve the process as required and, ultimately, provide healthcare solutions that will optimally benefit patients.

## Author Contributions

NC initiated and led all the research studies referenced in this manuscript. A-PH, NC, and JC conceptualized, drafted, reviewed and edited the manuscript. All authors read and approved the final version of the manuscript.

### Conflict of Interest Statement

NC is employed by Novartis Pharma AG, JC by Novartis Pharmaceuticals Corp. (JC); A-PH is employed by Health Outcomes Strategies, LLC.

## Footnotes

^1^Conjoint analysis is a de-composition method, in that the implicit values for an attribute of an intervention are derived from some overall score for a profile consisting (conjointly) of two or more attributes (82).
